# Field emission from non-uniform carbon nanotube arrays

**DOI:** 10.1186/1556-276X-8-319

**Published:** 2013-07-10

**Authors:** Fernando F Dall'Agnol, Daniel den Engelsen

**Affiliations:** 1 Center for Information Technology Renato Archer CTI, Rod., D. Pedro I km 143.6, Amarais, Campinas, Sao Paulo 13069-901, Brazil

**Keywords:** Non-uniform array, Carbon nanotube array, CNT array, Field emitter array, Field emitter morphology, Field emission simulation

## Abstract

Regular arrays of carbon nanotubes (CNTs) are frequently used in studies on field emission. However, non-uniformities are always present like dispersions in height, radius, and position. In this report, we describe the effect of these non-uniformities in the overall emission current by simulation. We show that non-uniform arrays can be modeled as a perfect array multiplied by a factor that is a function of the CNTs spacing.

## Background

Carbon nanotube (CNT) arrays for field emission (FE) applications have been extensively studied experimentally and theoretically [1-5]. Various improvements to fabricate well-aligned CNT arrays have been achieved, but non-uniformities are always present. To build precise arrays is expensive and difficult in extending to large areas. Simulation of CNT arrays is cost effective; however, simulation of these structures including non-uniformity is rare in the literature. To model non-uniformities in FE, it is necessary to understand their effects on the emission current. The simulation of FE in large domains is notoriously difficult especially in three dimensions, which is necessary in this analysis. The difficulties include long simulation times, large computer memory requirements, and computational instability. The first analysis of this kind is the recent work of Shimoi and Tanaka [6]. They managed to perform three-dimensional (3D) simulations based on boundary elements that avoided meshing the volume of the 3D domain. They simulated carbon nanofibers with random position and height to match the emission pattern that they obtained experimentally. In this work, we perform simulations of non-uniform CNTs with dispersions in height, radius, and position in limited ranges and with small CNT aspect ratios aiming to correlate the current from non-uniform arrays with the current expected from perfect arrays. We restrict our analysis to a hemisphere-on-a-post model [4,6-8], in which the CNTs are regarded as perfect conductors, with a smooth surface and oriented normal to the substrate. In this report, we shall refer to these idealized tubes as CNTs.

## Methods

**Figure 1:**
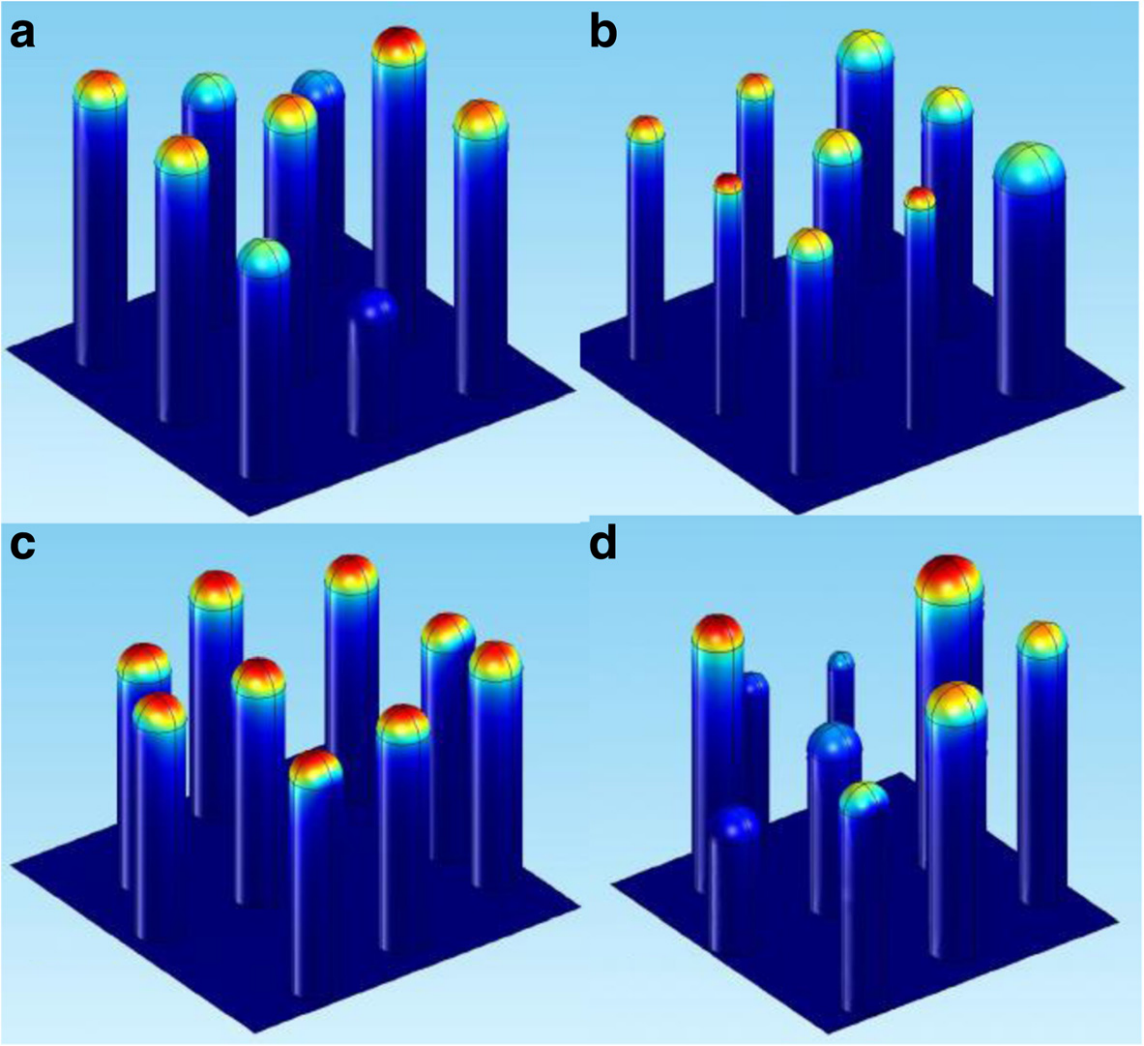
**Hemisphere-on-a-post model for a 3 × 3 non-uniform array domain.** In **(a)**, **(b)**, and **(c)**, respectively, height, radius, and position are separately randomized. In **(d)**, all three parameters are randomized at the same time. The red regions indicate strong electric field.

The CNTs are positioned in a 3 × 3 square array, as shown in Figure 1. We shall explain hereafter that a 3 × 3 square array is an efficient way to perform the simulations. The *i*th CNT height *H*_*i*_, radius *R*_*i*_, and coordinates (*X*_*i*_,*Y*_*i*_) are stochastic variables with expected values (or averages), respectively, equal to *h* = 10 a.u., *r* = 1 a.u., and (*x*_*i*_,*y*_*i*_) being the center of the *i*th unit cell in the array. Thus, the default aspect ratio is 10, which is quite small. However, larger aspect ratios cause simulation difficulties that will be discussed later. Despite this limitation, we think that the results provide a meaningful insight on the behavior of the current. We simulated aspect ratios up to 100 in graphenes randomizing only the positions. The results vary at most 25%, tending to increase slowly in a logarithmic pace as a function of aspect ratio. A complete analysis of graphene sheets will be presented in a forthcoming paper. The stochastic variables in our study will be limited to the following ranges:

(1) $$h\left(1-\frac{\alpha_{h}}{2}\right)<H_{i}<h\left(1+\frac{\alpha_{h}}{2}\right),$$

(2) $$r\left(1-\frac{\alpha_{r}}{2}\right)<R_{i}<r\left(1+\frac{\alpha_{r}}{2}\right),$$

and

(3) $$\left(x_{i}-\frac{\alpha_{p}}{2}s,y_{i}-\frac{\alpha_{p}}{2}s\right)<\left(X_{i},Y_{i}\right)<\left(x_{i}+\frac{\alpha_{p}}{2}s,y_{i}+\frac{\alpha_{p}}{2}s\right),$$

where *s* is the array spacing; *α*_*h*_, *α*_*r*_, and *α*_*p*_ can be interpreted as the range in percentage of the expected value. For instance, *α*_*h*_ = 1 implies that the height of the CNT can vary 100%, from 0.5 *h* to 1.5 *h*. The choice for these dispersion ranges was based on microscopic observations [6,9,10]. If *α* = 0, the corresponding stochastic variable is constant. Equation (3) states that the displacement range of the CNTs can vary from no displacement (*α*_*p*_ = 0) to displacements as large as half the length of the unit cell (*α*_*p*_ = 1). We analyze the emission current as a function of *s* from near close packed (*s* ≥ 0.25 *h*) to *s* = 10 *h* (approximately isolated tubes). The field enhancement and the screening effects are illustrated in Figure 1. In Figure 1a, only the heights are randomized. The taller the tube, the larger the field strength at the tip, represented in shades of red; shorter tubes are shielded. In Figure 1b, only the radii are randomized. The screening effect is approximately the same for all tubes, but the field enhancement is larger at the thinner ones. In Figure 1c, only the positions are randomized. In this case, some tubes are more screened than others depending on how they clump up, notice however, that the field strength at the tips are more homogeneous compared to Figure 1a,b. Indeed, the overall current is less affected by randomized positions than heights or radii for the separation shown in this figure. In Figure 1d, all variables are randomized at the same time. The CNTs are not allowed to overlap.

The simulations are performed using software COMSOL® v.4.2a, which is based on the finite elements method. The CNT array, as shown in Figure 1, is regarded as purely electrostatic system. A macroscopic vertical electric field of 10 GV/m is applied on the domain. The side boundaries have *symmetry boundary condition*, which states that there is no electric field perpendicular to these boundaries (**E**.**n** = 0) making them act as mirrors. These conditions determine the norm of the electric field in the domain.

**Figure 2:**
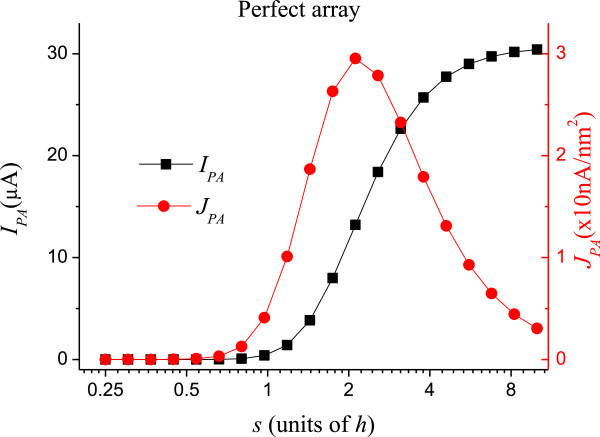
**Field emission current *I***_***PA***_**and current density *J***_***PA***_**of a perfect array.** The lattice spacing *s* is expressed in units of the CNT height *h*. The aspect ratio of the CNTs is 10 in this figure.

The local current density, *j*, is evaluated using Fowler Nordheim equation [11,12]:

(4) $$j=\frac{AE^{2}}{\varphi }\exp\left(-\frac{B\varphi^{3/2}}{E}\right),$$

where *A* = 1.56 × 10^-6^A eV V^-2^, *B* = 6.83 × 10^9^ eV^-3/2^ V/m, *ϕ* is the work function (in eV), and *E* is the local electric field (in V/m) at the surface of the CNTs. We use a work function of 5 eV for the CNTs. Equation (4) is integrated over the CNT's surfaces to obtain the overall current, which is normalized by the current from a perfect array *I*_*PA*_. Figure 2 shows *I*_*PA*_ and the overall current density, *J*_*PA*_, defined as the total current divided by the area of the array. The peak in *J*_*PA*_ at *s* ≅ 2 *h* indicates the ideal spacing for FE applications [13,14]. Note that *J*_*PA*_ is relatively small for *s* < *h*, so we shall focus most of our analyses to the region where *s* > *h*. The currents and current densities shown in Figure 2 for the perfect uniform lattice and uniform CNTs will be used to normalize the currents for the non-uniform structures.

Each simulation run, identified with the number of the run, *k*, has a particular set of randomized parameters that yield the normalized current, *I*_*k*_. The *I*_*k*_ values from a 3 × 3 domain present large variations, but after averaging 25 simulation runs, we obtain a smoother behavior, which is the expected values of the stochastic *I*_*k*_. The error in *I*_*k*_ decreases by a factor of 1/√*k*. In FE experiments, the observed current is the average over a large number of CNTs. We did 25 simulation runs of 3 × 3 CNTs, which is physically similar to simulate 225 CNTs in one run. However, the latter calculation is impossible due to memory and numerical instability. Even a 3 × 3 array takes a rather long time to simulate, and some of our results were not reliable at large spacing. We simulated arrays with 1 × 1, 2 × 2, 3 × 3, and 4 × 4 randomized CNTs. The average current depends on the size of the domain, but the convergence is fast. The normalized currents as a function of the spacing for 3 × 3 and 4 × 4 arrays are exactly the same within the error. Hence, a 3 × 3 domain is already large enough to represent a random field of CNTs.

## Results and discussion

**Figure 3:**
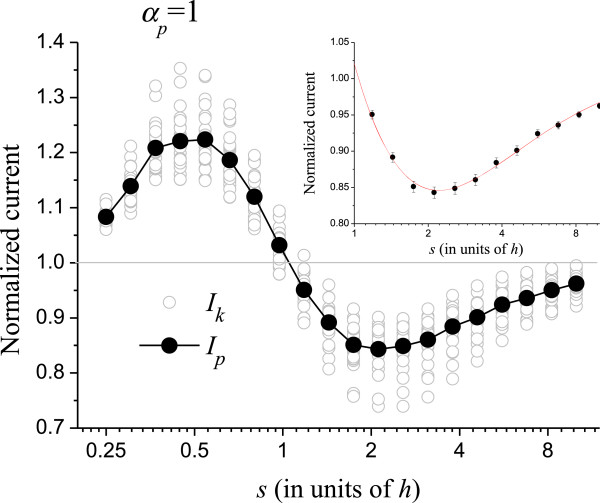
**Randomization in the (*x*,*y*) coordinates of the CNTs in the array.** The gray opened circles are the normalized current *I*_*k*_ from an individual simulation run. The full circles are the average over 25 runs (*I*_*p*_). The inset shows *s* > *h* superposed to an interpolating function that provides a numerical value for *I*_*p*_.

Figure 3 shows the result when only the positions of the CNTs are randomized (*α*_*p*_ = 1, *α*_*r*_ = *α*_*h*_ = 0). The normalized average *I*_*p*_ = <*I*_*k*_ > is shown in full circles. The gray line at *I*_*p*_ = 1 is drawn to guide the eye. The sine-like behavior of *I*_*p*_ is a consequence of the step shape of *I*_*PA*_ (see Figure 2), which increases fast at small *s* and saturates for *s* → *∞*. The random positioning causes some CNTs to lump, while others form a sparser configuration. At small *s*, the field enhancement of the slightly isolated CNTs dominates the lumping of CNTs elsewhere, thus *I*_*p*_ > 1. On the other hand, for large *s*, the CNTs are practically isolated, and their field enhancement of the CNTs is almost at a threshold value. In this case, the current from isolated CNTs is almost constant, while the screening effect of the lumped regions significantly reduces the current, so *I*_*p*_ < 1. For *s* → *∞*, the emitters are isolated, and it is unlikely that two or more emitters will become close enough to screen each other after random displacements; therefore, *I*_*p*_ tends to unity. At *s* ≅ *h*, field enhancement and screening on the randomized tubes compensate exactly and *I*_*p*_ = 1. At this point, misplaced CNTs do not affect the overall current expected from a perfect array. The inset in the figure shows the region for *s* > 1, which is the important region for FE applications as mentioned. We fitted this region with the simplest interpolating function to provide a numerical value for *I*_*p*_. The fitting curve is shown in the inset.

**Figure 4:**
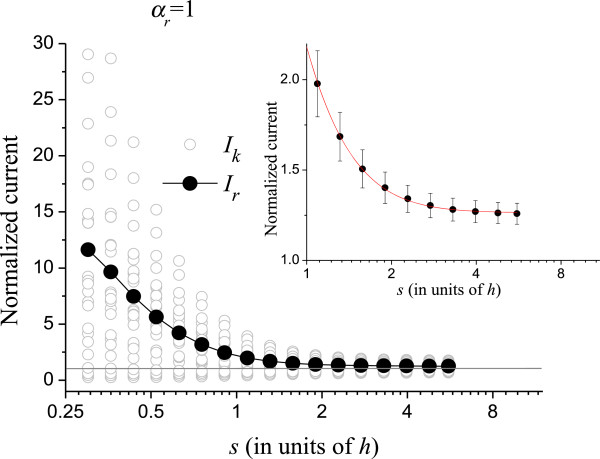
Normalized current from randomized radii of the CNTs.

**Figure 5:**
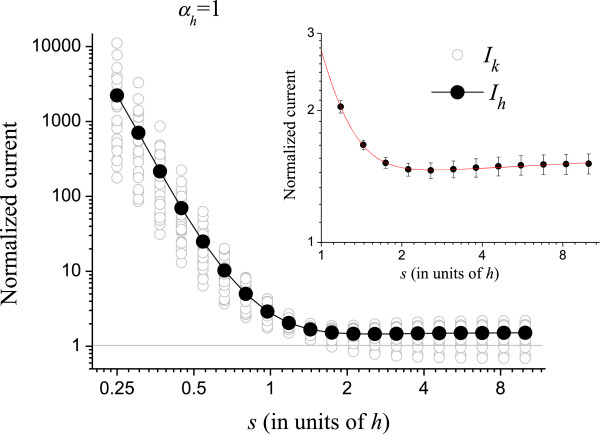
Normalized current from randomized heights of the CNTs.

Figures 4 and 5 show the normalized currents *I*_*r*_ and *I*_*h*_ for *α*_*r*_ = 1 and *α*_*h*_ = 1, respectively. Like in Figure 3, the horizontal axes in these figures are logarithmic. At small *s*, *I*_*r*_, and *I*_*h*_ are sensitive to the randomization as can be seen. In this region, fluctuations in height and radius largely decrease the electrostatic shielding as compared to the uniform CNTs, thus the normalized current becomes very high. It should be remembered that, although the normalized *I*_*r*_ and *I*_*h*_ are high for small *s*, the absolute current is actually very small, as can be seen in Figure 2. The insets show the curves for *s* > *h*. The interpolating functions used in Figures 3, 4, and 5 for *s* > *h* are

(5) $$I_{p}\left(s>h\right)=1.02-1.9{\left(s/h+1.01\right)}^{-1.845}\ln\left(s/h\right),$$

(6) $$I_{r}\left(s>h\right)=1.26+17.7{\left(s/h+0.833\right)}^{-4.884},$$

(7) $$I_{h}\left(s>h\right)=1.41+579{\left(s/h+1\right)}^{-8.766}+0.0477\ln\left(s/h\right).$$

Equations (5) to (7) have no physical meaning; they are mere interpolating functions only to provide numerical values between the simulated points. These interpolating functions were chosen for representing the shape of the curves by taking the logarithmic scale of the *x*-axis into account.

**Figure 6:**
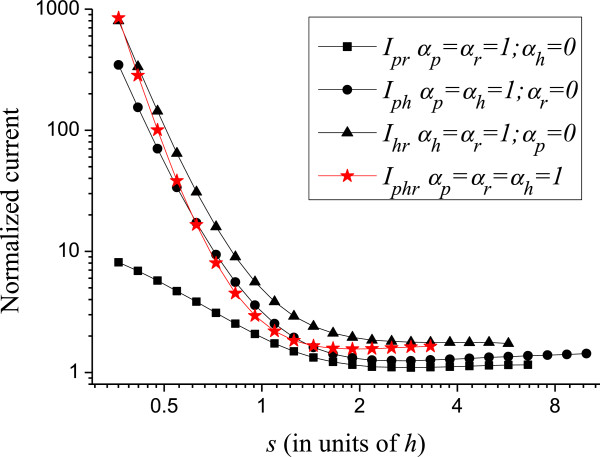
Normalized emission randomizing variables two at a time and all three variables simultaneously.

**Figure 7:**
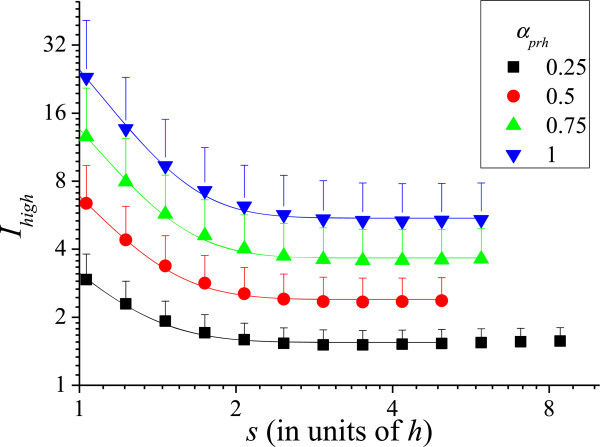
**Highest normalized emission *I***_**high **_**and the standard deviation *σI***_**high **_**as a function of the spacing.** The *σI*_high_ is shown as half error bars. These parameter can be used to estimate the fraction of CNTs that will burn out at certain current given the degree of non-uniformity.

Next, we analyze the effect of randomizing two parameters simultaneously. It is not trivial to evaluate, for example, *I*_*pr*_ knowing the values of *I*_*p*_ and *I*_*r*_. The difficulties are the non-linearity of Eq. (4) and the complicated local electric field *E* that appears in it. This field is a function of *X*_*i*_, *Y*_*i*_, *R*_*i*_ and *H*_*i*_ and does not have an analytic solution. Therefore, for this analysis, we need to vary two parameters simultaneously. Just as for *I*_*p*_, *I*_*r*_ or *I*_*h*_, the simulations are averaged over 25 runs. The results are shown in Figure 6. In this figure, the expected values of the normalized current are specified with two sub-indices that indicate the parameters that are varying. Figure 6 also shows the expected normalized current *I*_*prh*_, when varying the three parameters: position (*x*,*y*), radius, and height at the same time. Interestingly, *I*_*prh*_ is below the curves for *I*_*hr*_ and *I*_*ph*_ in some regions. This means that randomizing two parameters affects the average current more than varying three parameters in these regions. The curves are always greater than unity, typically between 1 and 4 for *s* > *h*. This is a consequence of randomization: some CNTs are less electrostatically screened causing them to surpass the emission of a perfect array. Furthermore, most CNTs are screened, as can be seen in Figure 1d; so, only few CNTs are accounting for the total current [6]. Then, by increasing the external electric field, these few CNTs will become overloaded before most CNTs can start contributing to the current. Consequently, the maximum current density of non-uniform arrays is limited by the current that these few CNTs can support. We define *I*_high_ as the highest CNT normalized current in the 3 × 3 array averaged over 100 runs. *I*_high_ comprehends 1/9 or 11.1% of the most emissive CNTs. Figure 7 shows *I*_high_ as a function of *s* for *s* > *h* and its standard deviation, *σI*_high_, shown in the figure as error bars. The *σI*_high_ can be used to determine what part of the CNTs is expected to burn in the non-uniform array given their tolerance, as we shall indicate below.

The interpolating functions for the curves of Figure 6 are

(8) $$I_{\mathrm{ph}}\left(s>h\right)=1.09+38.2{\left(s/h+0.6\right)}^{-6.235}+0.148\ln\left(s/h\right),$$

(9) $$I_{\mathrm{pr}}\left(s>h\right)=0.93+6.08{\left(s/h+0.72\right)}^{-3.19}+0.09\ln\left(s/h\right),$$

(10) $$I_{\mathrm{hr}}\left(s>h\right)=1.75+2.68{\left(s/h+0.033\right)}^{-4.21},$$

(11) $$I_{\mathrm{phr}}\left(s>h\right)=1.31+0.5{\left(s/h-0.23\right)}^{-3.634}+0.28\ln\left(s/h\right).$$

Equations (5) to (11) are valid for *α* = 1; however, our simulation results (not shown here) indicate that a quadratic function fits intermediate values 0 < *α* < 1 reasonably well. The following example gives a procedure to obtain the normalized current for any set (*α*_*p*_,*α*_*r*_,*α*_*h*_), with normalized current *I*(*α*_*p*_,*α*_*r*_,*α*_*h*_). In the simplest example, if only *α*_*p*_ varies, then

(12) $$I\left(\alpha_{p},0,0\right)=1+\alpha_{p}^{2}\left(I_{p}-1\right),$$

where *I*_*p*_ is given by Eq. (5). In another example, in which *α*_*p*_ and *α*_*r*_ are varying, then

(13) $$I\left(\alpha_{p},\alpha_{r},0\right)=I\left(\alpha_{p},0,0\right)+\alpha_{r}^{2}\left(I_{\mathrm{pr}}-I\left(\alpha_{p},0,0\right)\right),$$

where *I*_*pr*_ is given in Eq. (9). Finally, if all *α* parameters vary, we have

(14) $$I\left(\alpha_{p},\alpha_{r},\alpha_{h}\right)=I\left(\alpha_{p},\alpha_{r},0\right)+\alpha_{h}^{2}\left(I_{\mathrm{phr}}-I\left(\alpha_{p},\alpha_{r},0\right)\right),$$

where *I*_*phr*_ is given in Eq. (11).

From the data shown in Figure 7, we derive the following interpolating functions

(15) $$I_{\mathrm{high}}\left(s>h\right)=1+1.12\alpha_{\mathrm{prh}}+3.34\alpha_{\mathrm{prh}}^{2}+19\alpha_{\mathrm{prh}}^{2}\exp\left(-\frac{s/h-1}{0.283}\right),$$

where, *α*_*prh*_ = max(*α*_*p*,_*α*_*r*,_*α*_*h*_) and

(16) $$\sigma I_{\mathrm{high}}\left(s>h\right)=0.16\alpha_{\mathrm{prh}}+2.42\alpha_{\mathrm{prh}}^{2}+16.1\alpha_{\mathrm{prh}}^{2}\exp\left(-\frac{s/h-1}{0.26}\right).$$

Equations (15) and (16) give an upper estimate of the maximum current carried by individual CNTs, as a function of our randomization parameter *α*_*prh*_.

The fraction of CNTs expected to burn out can be evaluated from a Gaussian distribution as:

(17) $$\begin{array}{c} \xi =\frac{11.1\%}{\sigma I_{\mathrm{high}}\sqrt{2\pi }}\int_{I_{\max}}^{\infty }\exp\left(-\frac{{\left(i-I_{\mathrm{high}}\right)}^{2}}{2\sigma I_{\mathrm{high}}^{2}}\right)\mathrm{di}\\ \;=\frac{1}{2}\left(1+\mathrm{erf}\left(\frac{I_{\mathrm{high}}-I_{\max}}{\sigma I_{\mathrm{high}}\sqrt{2}}\right)\right)\times 11.1\%, \end{array}$$

where erf(*z*) is the error function, *I*_max_ is the normalized burn out current (or tolerance). The factor 11.1% is because Eqs. (15) and (16) account only for 1/9th of the CNTs in the 3 × 3 array.

Let us give an example: consider a non-uniform array with *α*_*p*_ = 0.4, *α*_*r*_ = 0.5, *α*_*h*_ =0.8 observed microscopically and *s* = 2 *h* yielding an average emission of 1 μA. From Eqs. (14), (15), and (16), we calculate a normalized current of *I* = 1.28, which corresponds to the 1 μA; *I*_high_ = 4.94 (3.86 μA) and *σI*_high_ = 1.90 (1.48 μA). Now, suppose *I*_max_ is 10 (7.81 μA), then the fraction *ξ* of emitters that will burn out at 1 μA is smaller than 0.04% according to Eq. (17). In this example, *I*_max_ is constant: otherwise, the calculation of *ξ* will be more elaborate. If *I*_max_ is a known function, then *ξ* must be integrated over *I*_max_ for a refined estimative. However, we shall not deepen our analysis on *ξ* in this paper.

## Conclusions

We simulated the behavior of the field emission current from non-uniform arrays of CNTs and obtained correction factors to multiply the current from a perfect CNT array toward the currents of non-uniform arrays. These correction functions are valid if the allowed dispersion in height and radius is kept inside the limits of 50% and 150% of their average values and if the randomization of the CNT position is done inside the designated unit cell. The uneven screening effect in non-uniform arrays causes many CNTs to become idle emitters while few may become overloaded and burn out. To avoid this, uniformity is desired: however, non-uniformities are always present in some degree, and our model describes how to treat them. This model can also be used in estimating how many CNTs are expected to burn given their tolerance and the total current extracted from the array.

We like to point out that in a previous work [15], we showed that the emission from 3D CNT arrays can be simulated in a two-dimensional (2D) rotationally symmetric system with proper boundary conditions. The currents from the 2D and 3D arrays are also related by a factor that is a function of the aspect ratio and spacing of the actual array. The combined correction factor from Eq. (14) and the procedure in [15] can considerably ease the modeling of FE from non-uniform CNT arrays, as they can be reduced to perfectly uniform arrays, which may be treated in a 2D model.

## Competing interests

Both authors declare that they have no competing interests.

## Authors' contributions

FFD did the simulations. FFD and DdE analyzed the results, discussed the models, and wrote the article. Both authors read and approved the final manuscript.
